# Supplementary material for: Agricultural land-uses consistently exacerbate infectious disease risks in Southeast Asia
Source: Nat Commun. 2019 Sep 20;10:4299. doi: 10.1038/s41467-019-12333-z (PMC6754503; doi:10.1038/s41467-019-12333-z)
Supplement: Supplementary file 1 — Supplementary Information [file 41467_2019_12333_MOESM1_ESM.pdf]

Agricultural land-uses consistently exacerbate infectious disease risks in Southeast Asia

Shah et al

Supplementary Information

Supplementary Note 1 - Example Search Strategy

Here we provide an example of the search strategy used for EMBASE:

1. zoonoses or zoonosis or infectio* or communicab* or emerg* or disease*

2. exp Communicable Diseases

3. South east Asia or SE Asia or Southeast Asia or Brunei or Cambodia or Indonesia or Laos or Malaysia or Myanmar or Philippines or Singapore or Thailand or Timor or Vietnam – TITLE AND ABSTRACT ONLY

4. exp Asia, Southeastern

5. 1 or 2

6. 3 or 4

7. land use* or land cover* or landscape* or habitat* or deforest* or agricultur* or farm* or urbani* or suburbani* or fragment*

8. 5 and 6 and 7

Google and Google Scholar were also used to search for published articles that may not have been indexed within the databases. The internet search engines typically returned several thousand results. Therefore, the searches were restricted to the first fifty hits and links to potentially relevant material were followed from the original hit. Finally, bibliographies of articles included in the review and previously published reviews were checked for references.

Supplementary Note 2 - Eligibility Criteria

Inclusion Criteria

- Geographical Location – Southeast Asia defined as Vietnam, Cambodia, Laos PDR, Thailand, Myanmar, Malaysia, Indonesia, Singapore, Philippines, East Timor and Brunei as part of the ASEAN region.
- Population – Adults in Southeast Asia aged 18 and above that work or live in or near agricultural land (NB – studies that assess total populations including both adult and children will be included).
- Type of exposure - Agricultural land use exposure was defined as any person who partakes in the cultivation of land and breeding of animals and plants to provide food, fibre, medicinal plants and other products to sustain and enhance either for domestic, residential, occupational or economic purposes.
- Type of comparator - No exposure to agricultural land use
- Types of outcome: Change in prevalence or incidence of infectious disease as a function of land use or land use change.
- Type of disease: All infectious diseases that are prevalent in humans in Southeast Asia with a biologically plausible link to land-use change including emerging, zoonotic, bacterial, viral, parasitic and vector-borne infections.
- Types of study – Peer reviewed empirical observational studies

Exclusion Criteria

- Articles based on non-communicable disease
- Articles based on infectious diseases of plants, invertebrates or fish
- Articles that do not study the impact of land use or land use change
- Articles that do not have a study context in SE Asia
- Articles not in English
- Theoretical research, reviews, commentaries or letters.
- Studies that presented odds ratios based on the co-infection of more than 1 disease
- Studies that assessed the impact of using human faeces (night soil) as fertiliser in agriculture
- Studies that assessed risk factors of disease in children

Duplicates were removed using reference management software (Endnote and Mendeley). If the inclusion of an article was in doubt in either the first two stages, the article was included, and the suitability determined at a later stage.

Supplementary Figure 1 - Meta-analysis of adjusted odds ratios

Author, Year	Exposure	Disease	Adjustment Type	aOR [95% CI]
Grigg et al, 2017	Plasmodium Knowlesi	Agriculture	Multivariate adjustment	1.89 [1.07, 3.34]
Hinjoy et al, 2012	Hepatitis E	Pig Farming	Multivariate adjustment	0.81 [0.49, 1.33]
Huong et al, 2015	Streptococcus Suis	Agriculture	Adjusted for sex, occupation, and eating practices	2.68 [1.52, 4.73]
Ngoc dinh et al, 2006	H5N1	Poultry Farming	Adjusted for age, sex, and location	3.00 [0.90, 10.00]
Nguyen et al, 2006	Hookworm	Agriculture	Multivariate adjustment including co-infection	2.20 [1.66, 2.91]
Ong et al, 2016	Opisthorchis Viverrini	Agriculture	Multivariate adjustment	0.44 [0.25, 0.78]
Pattasin et al, 2012	Malaria	Rubber Plantation	Multivariate adjustment	2.30 [1.03, 5.14]
Pham Duc et al, 2011	Entamoeba histolytica	Agriculture	Adjusted for age and sex	0.80 [0.30, 2.17]
Pham Duc et al, 2013	A. lumbricoides	Agriculture	Adjusted for age, sex, and season	1.50 [1.09, 2.07]
Pham Duc et al, 2014	Intestinal Enteric Disease	Agriculture	Multivariate adjusment including age and sex	1.10 [0.60, 2.01]
Rangsin et al, 2009	Opisthorchis Viverrini	Agriculture	Adjusted for sex, education level, and occupation	0.90 [0.49, 1.66]
Ridzuan et al, 2016	Leptospirosis	Cow Farming	Multivariate adjustment	4.78 [2.76, 8.27]
Ross et al, 2016	Any Helminth	Agriculture (Occupation)	Multivariate adjustment	1.47 [1.13, 1.91]
Rukmanee et al, 2008	Hookworm	Agriculture	Multivariate adjustment	1.08 [0.78, 1.49]
Satitvipawee et al , 2012	Malaria	Rubber Plantation	Adjusted by age and sex	1.06 [0.45, 2.49]
Tarafder et al, 2006	Schistosoma Japonicum	Agriculture	Multivariate adjustment	1.32 [0.84, 2.07]
Vallee et al, 2010	Orientia tsutsugamushi	Agriculture	Multivariate adjustment	2.10 [1.02, 4.30]
RE Model Pre Fill and Trim (Q = 66.09, df = 16, p = 0.000; I^2 = 80.3%)				1.46 [1.11, 1.92]
RE Model Post Fill and Trim (Q = 66.09, df = 16, p = 0.00; I^2 = 80.3%)				1.46 [1.11, 1.92]

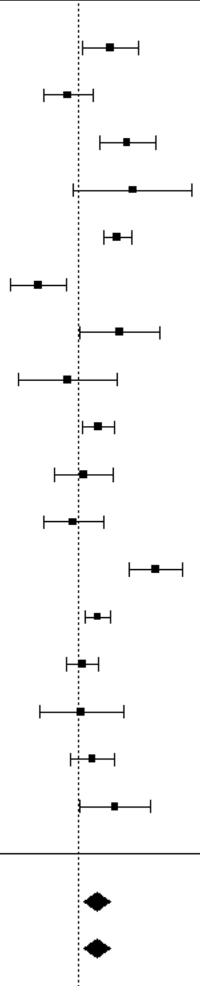

Figure 1 Adjusted meta-analysis of mutually exclusive risk estimates to determine the impact of within study confounding on the association between occupational or residential exposure to agricultural land use and infectious disease prevalence. Agriculture (Non-specific) is defined as a category where a person indicates they work or live in or near agriculture regardless of the type of agriculture. Multivariate adjustment is defined as adjustment for multiple factors which are not listed in the original study. A Z test was conducted to calculate p values. Square points show the crude odds ratio for each study, solid diamonds show the pooled meta-analysis estimate and error bars are defined as the 95% confidence interval

Note: Q, the Cochrane Q test. Df, degrees of freedom. p, p value. I2, test for heterogeneity. RE, random effects.

Supplementary Table 1 – Exposure Based Subgroup Analysis Results

Exposure Subgroups	Disease Subclass	No of Studies	OR	CI Low	CI High	P Value	E Value	Q Test	P Value	I^2
Bovine Exposure	All Diseases	3	2.09	0.80	5.49	0.130	2.96	15.96	<0.001	84.51
Bovine Exposure	Bacterial	2	2.40	0.57	10.12	0.230	3.39	15.39	<0.001	93.50
Bovine Exposure	Vector-Borne	3	2.09	0.80	5.49	0.130	2.96	15.96	<0.001	84.51
Bovine Exposure	Zoonotic	3	2.09	0.80	5.49	0.130	2.96	15.96	<0.001	84.51
Livestock exposure	All Diseases	8	2.54	1.37	4.72	<0.001	3.57	32.25	<0.001	76.02
Livestock exposure	Bacterial	5	4.47	1.30	15.39	0.020	6.05	18.61	<0.001	88.05
Livestock exposure	Vector-Borne	6	2.52	1.48	4.28	<0.001	3.55	14.04	0.020	60.55
Livestock exposure	Viral	3	1.55	0.83	2.91	0.170	2.17	4.98	0.080	58.51
Livestock exposure	Zoonotic	8	2.46	1.35	4.48	<0.001	3.46	33.79	<0.001	77.23
Non-specific Agriculture	All Diseases	21	1.71	1.38	2.13	<0.001	2.42	76.09	<0.001	85.94
Non-specific Agriculture	Bacterial	5	1.79	0.97	3.31	0.060	2.53	27.05	<0.001	89.41
Non-specific Agriculture	Parasitic	16	1.74	1.41	2.13	<0.001	2.45	47.65	<0.001	82.77
Non-specific Agriculture	Vector-Borne	7	1.85	1.18	2.90	0.010	2.61	32.40	<0.001	88.83
Non-specific Agriculture	Zoonotic	15	1.63	1.19	2.24	<0.001	2.30	61.69	<0.001	84.30
Oil Palm Plantation	All Diseases	2	3.25	2.29	4.61	<0.001	4.51	0.16	0.690	0.00
Oil Palm Plantation	Vector-Borne	2	3.25	2.29	4.61	<0.001	4.51	0.16	0.690	0.00
Oil Palm Plantation	Zoonotic	2	3.25	2.29	4.61	<0.001	4.51	0.16	0.690	0.00
Porcine Exposure	All Diseases	7	3.57	0.84	15.23	0.090	4.92	33.97	<0.001	95.59
Porcine Exposure	Bacterial	3	3.08	0.26	35.92	0.370	4.29	8.88	0.010	95.07
Porcine Exposure	Vector-Borne	5	3.09	0.58	16.46	0.190	4.29	23.80	<0.001	94.90
Porcine Exposure	Viral	4	4.31	0.49	37.81	0.190	5.85	24.62	<0.001	96.27
Porcine Exposure	Zoonotic	7	3.57	0.84	15.23	0.090	4.92	33.97	<0.001	95.59
Poultry Exposure	All Diseases	2	0.91	0.24	3.45	0.890	0.00	6.59	0.010	84.83
Poultry Exposure	Vector-Borne	2	0.91	0.24	3.45	0.890	0.00	6.59	0.010	84.83
Poultry Exposure	Zoonotic	2	0.91	0.24	3.45	0.890	0.00	6.59	0.010	84.83
Rice Paddy	All Diseases	5	1.34	0.81	2.23	0.250	1.84	21.78	<0.001	87.15
Rice Paddy	Bacterial	3	1.40	0.71	2.77	0.330	1.93	10.83	<0.001	82.33
Rice Paddy	Vector-Borne	4	1.17	0.62	2.21	0.620	1.54	14.73	<0.001	80.86
Rice Paddy	Zoonotic	4	1.17	0.62	2.21	0.620	1.54	14.73	<0.001	80.86
Rubber Plantation	All Diseases	5	2.27	1.82	2.82	<0.001	3.20	1.33	0.860	0.00
Rubber Plantation	Bacterial	2	2.27	1.79	2.89	<0.001	3.21	0.04	0.830	0.00
Rubber Plantation	Parasitic	3	2.24	1.35	3.74	<0.001	3.17	1.28	0.530	0.00
Rubber Plantation	Vector-Borne	5	2.27	1.82	2.82	<0.001	3.20	1.33	0.860	0.00
Rubber Plantation	Zoonotic	3	2.31	1.83	2.94	<0.001	3.27	0.99	0.610	0.00

Supplementary Table 2 – Exposure Based Subgroup Publication Bias Test Results

			Original Meta-Analysis				Fill and Trim Test				Eggers Test	
Exposure Subgroups	Disease Subclass	No of Studies	OR	CI Low	CI High	P Value	OR (FT)	P Value	CI Low (FT)	CI High (FT)	Eggers T Statistic	P Value
Bovine Exposure	All Diseases	3	2.09	0.80	5.49	0.130	2.09	0.134	0.80	5.49	-0.06	0.9645
Bovine Exposure	Vector-Borne	3	2.09	0.80	5.49	0.130	2.09	0.134	0.80	5.49	-0.06	0.9645
Bovine Exposure	Zoonotic	3	2.09	0.80	5.49	0.130	2.09	0.134	0.80	5.49	-0.06	0.9645
Livestock exposure	All Diseases	8	2.54	1.37	4.72	<0.001	1.62	0.209	0.76	3.43	1.33	0.2324
Livestock exposure	Bacterial	5	4.47	1.30	15.39	0.020	2.31	0.274	0.51	10.39	0.86	0.4518
Livestock exposure	Vector-Borne	6	2.52	1.48	4.28	<0.001	2.52	0.001	1.48	4.28	0.13	0.9063
Livestock exposure	Viral	3	1.55	0.83	2.91	0.170	0.98	0.963	0.49	1.98	3.59	0.1731
Livestock exposure	Zoonotic	8	2.46	1.35	4.48	<0.001	1.96	0.072	0.94	4.09	1.58	0.1659
Non-specific Agriculture	All Diseases	21	1.71	1.38	2.13	<0.001	1.48	0.001	1.18	1.86	-0.88	0.3904
Non-specific Agriculture	Bacterial	5	1.79	0.97	3.31	0.060	1.79	0.062	0.97	3.31	-0.11	0.9222
Non-specific Agriculture	Parasitic	16	1.74	1.41	2.13	<0.001	1.62	<0.001	1.32	1.99	-0.85	0.4088
Non-specific Agriculture	Vector-Borne	7	1.85	1.18	2.90	0.010	1.85	0.008	1.18	2.90	0.34	0.7471
Non-specific Agriculture	Zoonotic	15	1.63	1.19	2.24	<0.001	1.37	0.060	0.99	1.92	-0.20	0.8422
Porcine Exposure	All Diseases	7	3.57	0.84	15.23	0.09	3.57	0.086	0.84	15.23	4.56	0.0061
Porcine Exposure	Bacterial	3	3.08	0.26	35.92	0.370	3.08	0.369	0.26	35.92	2.21	0.2700
Porcine Exposure	Vector-Borne	5	3.09	0.58	16.46	0.190	3.09	0.187	0.58	16.46	3.69	0.0346
Porcine Exposure	Viral	4	4.31	0.49	37.81	0.190	4.31	0.187	0.49	37.81	4.00	0.0572
Porcine Exposure	Zoonotic	7	3.57	0.84	15.23	0.090	3.57	0.086	0.84	15.23	4.56	0.0061
Rice Paddy	All Diseases	5	1.34	0.81	2.23	0.250	1.81	0.037	1.04	3.17	-1.58	0.2129
Rice Paddy	Bacterial	3	1.40	0.71	2.77	0.330	1.40	0.333	0.71	2.77	-0.14	0.9107
Rice Paddy	Vector-Borne	4	1.17	0.62	2.21	0.620	1.40	0.285	0.76	2.57	-0.70	0.5571
Rice Paddy	Zoonotic	4	1.17	0.62	2.21	0.620	1.40	0.285	0.76	2.57	-0.70	0.5571
Rubber Plantation	All Diseases	5	2.27	1.82	2.82	<0.001	2.27	<0.001	1.82	2.82	0.33	0.7632
Rubber Plantation	Parasitic	3	2.24	1.35	3.74	<0.001	1.84	0.004	1.21	2.78	4.41	0.1420
Rubber Plantation	Vector-Borne	5	2.27	1.82	2.82	<0.001	2.27	<0.001	1.82	2.82	0.33	0.7632
Rubber Plantation	Zoonotic	3	2.31	1.83	2.94	<0.001	2.31	<0.001	1.83	2.94	0.62	0.6473

Supplementary Table 3 – Disease Subgroup Analysis

Disease	No of Studies	Original Meta-Analysis				I^2	E Value	Fill and Trim Test				Eggers Test	
		OR	CI Low	CI High	P Value			OR (FT)	P Value	CI Low (FT)	CI High (FT)	Eggers T Statistic	p value
Ascaris lumbricoides	4	1.27	0.59	2.73	0.540	96.52	1.71	1.49	0.344	0.68	3.06	-1.45	0.28
Entamoeba histolytica	3	1.01	0.73	1.4	0.930	0	1.13	1.01	0.945	0.73	1.40	0.01	0.99
Giardia intestinalis	2	0.51	0.17	1.52	0.230	39	0	NA	NA	NA	NA	NA	NA
Hookworm	3	2.42	1.56	3.75	<0.001	91.96	3.41	2.42	<0.001	1.56	3.75	1.20	0.44
Leptospirosis	4	1.36	0.55	3.32	0.500	90.6	1.86	1.71	0.204	0.75	3.93	-0.16	0.88
Malaria	5	2	1.46	2.73	<0.001	46.02	2.83	1.57	0.007	1.13	2.19	1.38	0.26
Opisthorchis viverrini	7	1.51	0.84	2.7	0.170	78.14	2.1	1.20	0.497	0.71	2.04	-0.22	0.83
Orientia tsutsugamushi	5	2.37	1.41	3.96	<0.001	85.83	3.34	2.37	0.001	1.41	3.96	-0.24	0.82
Rickettsia typhi	2	1.12	0.5	2.5	0.780	0	1.42	NA	NA	NA	NA	NA	NA
Schistosoma japonicum	2	1.71	1.18	2.48	<0.001	63.92	2.41	NA	NA	NA	NA	NA	NA
Spotted fever group	2	3.91	2.61	5.85	<0.001	55.23	5.35	NA	NA	NA	NA	NA	NA
Trichuris trichuria	4	1.4	1.27	1.53	<0.001	0	1.93	1.41	<0.001	1.29	1.55	-1.25	0.34

Supplementary Note 3 - References of Included Studies

1. Chaiputcha K, Promthet S, Bradshaw P. 2015. Prevalence and risk factors for infection by opisthorchis viverrini in an urban area of Mahasarakham province, Northeast Thailand. Asian Pacific J Cancer Prev 16:4173–4176; doi:10.7314/APJCP.2015.16.10.4173.
2. Chan BTE, Amal RN, Noor Hayati MI, Kino H, Anisah N, Norhayati M, et al. 2008. Seroprevalence of toxoplasmosis among migrant workers from different Asian countries working in Malaysia. Southeast Asian J Trop Med Public Health 39: 9–13.
3. Chudthaisong N, Promthet S, Bradshaw P. 2015. Risk factors for Opisthorchis viverrini Infection in Nong Khai Province, Thailand. Asian Pacific J Cancer Prev 16:4593–4596; doi:10.7314/APJCP.2015.16.11.4593.
4. Thi T, Dao H, Bui T Van, Nji E, Gabriël S, Thi T, et al. 2016. Acta Tropica Opisthorchis viverrini infections and associated risk factors in a lowland area of Binh Dinh Province , Central Vietnam. Acta Trop 157:151–157; doi:10.1016/j.actatropica.2016.01.029.
5. Erhart A, Ngo DT, Phan VK, Ta TT, Van Overmeir C, Speybroeck N, et al. 2005. Epidemiology of forest malaria in central Vietnam: a large scale cross-sectional survey. Malar J 4:58; doi:10.1186/1475-2875-4-58.
6. Fuhrimann S, Winkler MS, Pham-Duc P, Do-Trung D, Schindler C, Utzinger J, et al. 2016. Intestinal parasite infections and associated risk factors in communities exposed to wastewater in urban and peri-urban transition zones in Hanoi, Vietnam. Parasites and Vectors 9:1–14; doi:10.1186/s13071-016-1809-6.
7. Grigg MJ, Cox J, William T, Jelip J, Fornace KM, Brock PM, et al. 2017. Individual-level factors associated with the risk of acquiring human Plasmodium knowlesi malaria in Malaysia: a case-control study. Lancet Planet Heal 1:e97–e104; doi:10.1016/S2542-5196(17)30031-1.
8. Hinjoy S, Nelson KE, Gibbons R V., Jarman RG, Mongkolsirichaikul D, Smithsuwan P, et al. 2013. A Cross-Sectional Study of Hepatitis E Virus Infection in Healthy People Directly Exposed and Unexposed to Pigs in a Rural Community in Northern Thailand. Zoonoses Public Health 60:555–562; doi:10.1111/zph.12030.
9. Huong VTL, Thanh L V, Phu VD, Trinh DT, Inui K, Tung N, et al. 2015. Temporal and spatial association of Streptococcus suis infection in humans and porcine reproductive and respiratory syndrome outbreaks in pigs in northern Vietnam. Epidemiol Infect 144:35–44; doi:10.1017/S0950268815000990.
10. Kaewpitoon S, Rujirakul R, Wakkuwattapong P, Matrakool L, Tongtawee T, Panpimanmas S, et al. 2016. Opisthorchis viverrini infection among people in the border areas of three provinces, northeast of thailand. Asian Pacific J Cancer Prev 17: 2973–2977.
11. Kaewpitoon S, Rujirakul R, Ueng-arporn N, Matrakool L, Namwichaisirikul N, Churproong S, et al. 2012. Community-Based cross-sectional study of carcinogenic human liver fluke in elderly from surin province, thailand. Asian Pacific J Cancer Prev 13:4285–4288; doi:10.7314/APJCP.2012.13.9.4285.
12. Kaewpitoon S, Kaewpitoon N, Rujirakul R, Ueng-Arporn N, Matrakool L, Tongtawee T. 2015. The Carcinogenic Liver Fluke Opisthorchis viverrini among Rural Community People in Northeast Thailand: a Cross- Sectional Descriptive Study using Multistage Sampling Technique. Asian Pac J Cancer Prev 16: 7803–7807.
13. Soraya Kaewpitoon, Ryan Loyd NK. 2015. A cross-sectional survey of intestinal helminthiases in rural communities of Nakhon Ratchasima Province, Thailand. J Med Assoc Thail 98: S27–S32.

14. Kawaguchi L, Sengkeopraseuth B, Tsuyuoka R, Koizumi N, Akashi H, Vongphrachanh P, et al. 2008. Seroprevalence of leptospirosis and risk factor analysis in flood-prone rural areas in Lao PDR. Am J Trop Med Hyg 78:957–961; doi:78/6/957 [pii].
15. Kho KL, Koh FX, Hasan LIM, Wong LP, Kisomi MG, Bulgiba A, et al. 2017. Rickettsial seropositivity in the indigenous community and animal farm workers, and vector surveillance in Peninsular Malaysia. Emerg Microbes Infect 6:e18; doi:10.1038/emi.2017.4.
16. Manin BO, Ferguson HM, Vythilingam I, Fornace K, William T, Torr SJ, et al. 2016. Investigating the Contribution of Peri-domestic Transmission to Risk of Zoonotic Malaria Infection in Humans. PLoS Negl Trop Dis 10:1–14; doi:10.1371/journal.pntd.0005064.
17. Dinh PN, Long HT, Tien NTK, Hien NT, Mai LTQ, Phong LH, et al. 2006. Risk factors for human infection with avian influenza A H5N1, Vietnam, 2004. Emerg Infect Dis 12:1841–1847; doi:10.3201/eid1212.060829.
18. Nguyen PH, Nguyen KC, Nguyen TD, Le MB, Bern C, Flores R, et al. 2006. Intestinal helminth infections among reproductive age women in Vietnam: Prevalence, co-infection and risk factors. Southeast Asian J Trop Med Public Health 37: 865–874.
19. Ong X, Wang YC, Sithithaworn P, Namsanor J, Taylor D, Laithavewat L. 2016. Uncovering the Pathogenic Landscape of Helminth (Opisthorchis viverrini) Infections: A Cross-Sectional Study on Contributions of Physical and Social Environment and Healthcare Interventions. PLoS Negl Trop Dis 10:1–21; doi:10.1371/journal.pntd.0005175.
20. Parashar UD, Sunn LM, Ong F, Mounts AW, Arif MT, Ksiazek TG, et al. 2000. Case-control study of risk factors for human infection with a new zoonotic paramyxovirus, Nipah virus, during a 1998-1999 outbreak of severe encephalitis in Malaysia. J Infect Dis 181:1755–1759; doi:10.1086/315457.
21. Pattanasin S, Satitvipawee P, Wongklang W, Viwatwongkasem C, Bhumiratana A, Soontornpipit P, et al. 2012. Risk factors for malaria infection among rubber tappers living in a malaria control program area in southern Thailand. Southeast Asian J Trop Med Public Health 43: 1313–1325.
22. Pham Duc P, Nguyen-Viet H, Hattendorf J, Zinsstag J, Dac Cam P, Odermatt P. 2011. Risk factors for Entamoeba histolytica infection in an agricultural community in Hanam province, Vietnam. Parasit Vectors 4:102; doi:10.1186/1756-3305-4-102.
23. Pham-Duc P, Nguyen-Viet H, Hattendorf J, Zinsstag J, Phung-Dac C, Zurbrügg C, et al. 2013. Ascaris lumbricoides and Trichuris trichiura infections associated with wastewater and human excreta use in agriculture in Vietnam. Parasitol Int 62:172–180; doi:10.1016/j.parint.2012.12.007.
24. Pham-Duc P, Nguyen-Viet H, Hattendorf J, Cam PD, Zurbrügg C, Zinsstag J, et al. 2014. Diarrhoeal diseases among adult population in an agricultural community Hanam province, Vietnam, with high wastewater and excreta re-use. BMC Public Health 14:978; doi:10.1186/1471-2458-14-978.
25. Ridzuan JM, Aziah BD, Zahiruddin WM. 2016. Work Environment- Related Risk Factors for Leptospirosis among Plantation Workers in Tropical Countries: Evidence from Malaysia. Num Int J Occup Env Med 77: 156–163.
26. Vallé J, Thaojaikong T, Moore CE, Phetsouvanh R, Richards AL, Souris M, et al. 2010. Contrasting spatial distribution and risk factors for past infection with scrub typhus and murine typhus in Vientiane city, Lao PDR. PLoS Negl Trop Dis 4:1–10; doi:10.1371/journal.pntd.0000909.
27. Wattanayingcharoenchai S, Nithikathkul C, Wongsaroj T, Royal L, Reungsang P. 2011. Geographic information system of Opisthorchis viverrini in northeast Thailand. Asian Biomed 5:687–691; doi:10.5372/1905-7415.0505.090.
28. Tarafder MR, Balolong E, Carabin H, Bélisle P, Tallo V, Joseph L, et al. 2006. A cross-sectional study of the prevalence of intensity of infection with Schistosoma japonicum in 50 irrigated and rain-fed villages in Samar Province, the Philippines. BMC Public Health 6:61; doi:10.1186/1471-2458-6-61.
29. Tee TS, Kamalanathan M, Suan KA, Chun SS, Ming HT, Yasin RM, et al. 1999. Seroepidemiologic survey of Orientia tsutsugamushi, Rickettsia typhi, and TT118 spotted fever group rickettsiae in rubber estate workers in Malaysia. Am J Trop Med Hyg 61:73–77; doi:10.4269/ajtmh.1999.61.73.
30. Dev S, Pahang J, Lumpur K. 2000. Transactionsoftheroyalsocietyoftropicalmedicineandhygient? (2000)94,280-284. 280–284.
31. Robertson ID. 2015. Seroepidemiological study of leptospirosis among the communities living in periurban areas of Sarawak Seroepidemiological study of leptospirosis among the communities living in periurban areas of Sarawak ,. 70: 288–294.

32. Tay ST, Mohamed Zan HA, Lim YAL, Ngui R. 2013. Antibody Prevalence and Factors Associated with Exposure to Orientia tsutsugamushi in Different Aboriginal Subgroups in West Malaysia. PLoS Negl Trop Dis 7; doi:10.1371/journal.pntd.0002341.
33. Ross AGP, Olveda RM, McManus DP, Harn DA, Chy D, Li Y, et al. 2017. Risk factors for human helminthiases in rural Philippines. Int J Infect Dis 54:150–155; doi:10.1016/j.ijid.2016.09.025.
34. Rukmanee P, Wuthisen P, Thanyavanich N, Puangsa-Art S, Rukmanee N. 2008. Factors associated with intestinal parasites among households in Ratchaburi Province, Thai-Myanmar border area. J Trop Med Parasitol 31: 85–94.
35. Satitvipawee P, Wongkhang W, Pattanasin S, Hoithong P, Bhumiratana A. 2012. Predictors of malaria-association with rubber plantations in Thailand. BMC Public Health 12:1115; doi:10.1186/1471-2458-12-1115.
36. Somboon P, Aramrattana A, Lines J, Webber R. 1998. Entomological and epidemiological investigations of malaria transmission in relation to population movements in forest areas of north-west Thailand. Southeast Asian J Trop Med Public Health 29: 3–9.
37. Tan DS. 1979. Leptospirosis in West Malaysia--epidemiology and laboratory diagnosis. Malays J Pathol 2: 1–6.
38. Rangsin R, Mungthin M, Taamasri P, Mongklon S, Aimpun P, Naaglor T, et al. 2009. Incidence and risk factors of Opisthorchis viverrini infections in a rural community in Thailand. Am J Trop Med Hyg 81:152–155; doi:81/1/152 [pii].
