# Supplementary material for: Agricultural land-uses consistently exacerbate infectious disease risks in Southeast Asia
Source: Nat Commun. 2019 Sep 20;10:4299. doi: 10.1038/s41467-019-12333-z (PMC6754503; doi:10.1038/s41467-019-12333-z)
Supplement: Supplementary file 3 — Reporting Summary [file 41467_2019_12333_MOESM3_ESM.pdf]

nature research

Corresponding author(s): *Double-blind peer review submissions: write DBPR and your manuscript number here instead of author names.*

Last updated by author(s): *YYYY-MM-DD*

# Reporting Summary

Nature Research wishes to improve the reproducibility of the work that we publish. This form provides structure for consistency and transparency in reporting. For further information on Nature Research policies, see Authors & Referees and the Editorial Policy Checklist .

## Statistics

For all statistical analyses, confirm that the following items are present in the figure legend, table legend, main text, or Methods section.

| n/a | Confirmed | |
|---|---|---|
| ☐ | ☒ | The exact sample size (*n*) for each experimental group/condition, given as a discrete number and unit of measurement |
| ☐ | ☒ | A statement on whether measurements were taken from distinct samples or whether the same sample was measured repeatedly |
| ☐ | ☒ | The statistical test(s) used AND whether they are one- or two-sided<br>*Only common tests should be described solely by name; describe more complex techniques in the Methods section.* |
| ☐ | ☒ | A description of all covariates tested |
| ☐ | ☒ | A description of any assumptions or corrections, such as tests of normality and adjustment for multiple comparisons |
| ☐ | ☒ | A full description of the statistical parameters including central tendency (e.g. means) or other basic estimates (e.g. regression coefficient) AND variation (e.g. standard deviation) or associated estimates of uncertainty (e.g. confidence intervals) |
| ☐ | ☒ | For null hypothesis testing, the test statistic (e.g. *F*, *t*, *r*) with confidence intervals, effect sizes, degrees of freedom and *P* value noted<br>*Give P values as exact values whenever suitable.* |
| ☒ | ☐ | For Bayesian analysis, information on the choice of priors and Markov chain Monte Carlo settings |
| ☒ | ☐ | For hierarchical and complex designs, identification of the appropriate level for tests and full reporting of outcomes |
| ☐ | ☒ | Estimates of effect sizes (e.g. Cohen's *d*, Pearson's *r*), indicating how they were calculated |

*Our web collection on statistics for biologists contains articles on many of the points above.*

## Software and code

Policy information about availability of computer code

| | |
|---|---|
| Data collection | Microsoft Excel was used for data extraction. |
| Data analysis | All data was extracted in Microsoft Excel. All statistical analyses were conducted in R version 3.2.5 51 with the “metafor” package. This manuscript was referenced using Mendeley 1.17.12. |

For manuscripts utilizing custom algorithms or software that are central to the research but not yet described in published literature, software must be made available to editors/reviewers. We strongly encourage code deposition in a community repository (e.g. GitHub). See the Nature Research guidelines for submitting code & software for further information.

## Data

Policy information about availability of data

All manuscripts must include a data availability statement. This statement should provide the following information, where applicable:

- Accession codes, unique identifiers, or web links for publicly available datasets
- A list of figures that have associated raw data
- A description of any restrictions on data availability

The authors declare that all published data collated during the systematic review supporting the findings of this study are available within the paper and its supplementary information files. The final dataset is presented in Supplementary Information Dataset 1 and Note 3. This dataset presents information extracted by the reviewers and highlights the estimates used for each analysis. A description of the dataset is presented in Description of Additional Supplementary Files.

# Field-specific reporting

Please select the one below that is the best fit for your research. If you are not sure, read the appropriate sections before making your selection.

# Ecological, evolutionary & environmental sciences study design

All studies must disclose on these points even when the disclosure is negative.

| | |
|---|---|
| Study description | We conducted a systematic review and meta-analysis to test and quantify the associations between occupational or residential exposure to agriculture/agricultural land use (e.g. whether people work or live in agriculture) and prevalence and incidence of human infectious disease, focusing on the highly diverse yet tractable model system of South-East Asia. |
| Research sample | Following PRISMA guidelines and the PICOS framework, we considered the following factors to determine eligibility criteria: 'study question', 'populations', 'exposure', 'comparators', and 'outcome'. A description of each follows.<br>Study Question - Is there an association between occupational or residential exposure to agricultural land uses and being infected with a pathogen for adults aged 18 and above in SE Asia?<br><br>Study Design – Empirical observational studies (longitudinal cohorts, case control or cross sectional) studies conducted in the Association of Southeast Asian Nations (ASEAN) region and reported in English were considered eligible. We anticipated that the extent and effects of language bias may have diminished recently because of the shift towards publication of studies in English; however, we reserved the option to have non-English articles translated to bolster sample sizes if a reasonable number of non-English studies were found.<br><br>Populations – This study drew participants from the general adult population aged 18 and above in SE Asia. Studies that recruited participants of all ages (including children) were also included. Studies that focused exclusively on the child population were excluded.<br>Exposure – The primary exposure of interest was defined as occupational or residential exposure to agriculture or agricultural land use. This was defined as whether study participants would be working or living in or near agricultural land. Specifically, agricultural exposure was defined as any person who partakes in the cultivation of land and breeding of animals and plants to provide food, fibre, medicinal plants and other products either for domestic, residential, occupational or economic purposes.<br>Comparators – Studies were included if they compared outcomes in the exposed group with those in a group of unexposed people (people who are not occupationally or residentially exposed to agriculture or agricultural land use).<br>Outcome – Studies were included if one of the primary outcomes include prevalence, seroprevalence or incidence for all infectious diseases that have a biologically plausible link to agriculture or agricultural land use.<br><br>Inclusion Criteria<br>• Geographical Location – Southeast Asia defined as Vietnam, Cambodia, Laos PDR, Thailand, Myanmar, Malaysia, Indonesia, Singapore, Philippines, East Timor and Brunei as part of the ASEAN region.<br>• Population – Adults in Southeast Asia aged 18 and above that work or live in or near agricultural land (NB – studies that assess total populations including both adult and children will be included).<br>• Type of exposure - Agricultural land use exposure was defined as any person who partakes in the cultivation of land and breeding of animals and plants to provide food, fibre, medicinal plants and other products to sustain and enhance either for domestic, residential, occupational or economic purposes.<br>• Type of comparator - No exposure to agricultural land use<br>• Types of outcome: Change in prevalence or incidence of infectious disease as a function of land use or land use change.<br>• Type of disease: All infectious diseases that are prevalent in humans in Southeast Asia with a biologically plausible link to land-use change including emerging, zoonotic, bacterial, viral, parasitic and vector-borne infections.<br>• Types of study – Peer reviewed empirical observational studies<br>Exclusion Criteria<br>• Articles based on non-communicable disease<br>• Articles based on infectious diseases of plants, invertebrates or fish<br>• Articles that do not study the impact of land use or land use change<br>• Articles that do not have a study context in SE Asia<br>• Articles not in English<br>• Theoretical research, reviews, commentaries or letters.<br>• Studies that presented odds ratios based on the co-infection of more than 1 disease<br>• Studies that assessed the impact of using human faeces (night soil) as fertiliser in agriculture<br>• Studies that assessed risk factors of disease in children<br>Duplicates were removed using reference management software (Endnote and Mendeley). If the inclusion of an article was in doubt in either the first two stages, the article was included, and the suitability determined at a later stage.<br><br>34 mutually exclusive studies were included in the regional meta-analysis and a total of 37 mutually exclusive studies were included in the multiple subgroup analyses. Studies spanned five countries ('Thailand' = 11, 'Malaysia' = 10, 'Vietnam' = 9, 'Philippines' = 2, 'Lao PDR' = 2), two designs ('cross-sectional' = 27, 'case-control' = 7) and were assessed as being of varying quality using two study quality tools (OHAT – definitely low risk of bias = 2, probably low risk of bias = 25, probably high risk of bias = 10 and NHLBI – good = 7, fair = 23, poor = 4). A total of 80 effect estimates were extracted consisting of 26 infectious diseases and 12 different exposures. All included studies were in English and no studies were found to be in any other language. |
| Sampling strategy | Following PRISMA protocol and reporting standards for systematic reviews, we independently and systematically screened articles in April 2017 using five academic literature databases: Medline, PubMed, Global Health, Web of Science and EMBASE alongside Google Scholar.<br>Search strings were created through a PECOS statement using three categories (exposure, location and outcome) with Boolean operators AND between categories and OR within categories. Where applicable, MeSH terms for communicable disease, SE Asia, land |

use and agriculture were also used. Differing land use types were incorporated into the search strategy to improve the sensitivity of the search. To improve the specificity of the search strategy, the location category was only applied for title and abstracts, to capture all publications that had a study context within SE Asia. No language restrictions were placed within the search strategy.

Here we provide an example of the search strategy used for EMBASE:
1. zoonoses or zoonosis or infectio* or communicab* or emerg* or disease*
2. exp Communicable Diseases
3. South east Asia or SE Asia or Southeast Asia or Brunei or Cambodia or Indonesia or Laos or Malaysia or Myanmar or Philippines or Singapore or Thailand or Timor or Vietnam – TITLE AND ABSTRACT ONLY
4. exp Asia, Southeastern
5. 1 or 2
6. 3 or 4
7. land use* or land cover* or landscape* or habitat* or deforest* or agricultur* or farm* or urbani* or suburbani* or fragment*
8. 5 and 6 and 7

Data collection

Articles were initially assessed for relevance first by title, as well as keywords if these were available, then by abstract and finally by full text. We simultaneously assessed the suitability of the studies retained after screening for full text analysis for their potential inclusion in meta-analyses, rejecting studies for which risk or odds estimates could not be calculated. Disagreements were resolved by consensus, and where no consensus was achieved a third investigator was consulted. One reviewer (HS) then extracted outcome and exposure data as well as data on population and study characteristics into a bespoke data extraction framework, which was then validated by a second reviewer (PH).

Timing and spatial scale

The systematic review was conducted in April 2017.

Data exclusions

Studies that investigated non-communicable disease or infectious diseases of plants, invertebrates or fish were excluded. We also excluded studies that were not based on SE Asia, did not include some form of land use as an exposure or study focus, were theoretical research papers, reviews, commentaries or letters, or were not published in English. Studies that presented odds ratios based on the co-infection of more than 1 disease were excluded as co-infection could increase susceptibility to other infectious diseases. Studies that assessed the impact of using human faeces (night soil) as fertiliser in agriculture were also excluded. This is because using human faeces as fertiliser was not considered a land use but rather a confounding behavioural activity. Studies that assessed risk factors of disease in children were also excluded as children may be exposed to agricultural work but may also be more susceptible to certain diseases. The exclusion criteria are:

• Articles based on non-communicable disease
• Articles based on infectious diseases of plants, invertebrates or fish
• Articles that do not study the impact of land use or land use change
• Articles that do not have a study context in SE Asia
• Articles not in English
• Theoretical research, reviews, commentaries or letters.
• Studies that presented odds ratios based on the co-infection of more than 1 disease
• Studies that assessed the impact of using human faeces (night soil) as fertiliser in agriculture
• Studies that assessed risk factors of disease in children

Reproducibility

We conducted subgroup analysis using a priori subgroups to assess reproducibility between different subgroups and to assess heterogeneity, confounding and effect modification. We performed a subgroup analysis to determine how robust the regional meta-analysis result would be to certain study characteristics using the estimates from the regional meta-analysis. Here we created a priori subgroups on study type, sampling strategy, study setting, outcome measurement, study quality, study country and the characteristics of the study population.

Subgroup analyses were conducted on common exposures stratified by aetiological agent (parasitic, viral, bacterial) and transmission mode (vector-borne, zoonotic) or specific disease or disease complex subgroups that had more than two mutually exclusive estimates available. In order to preserve sample sizes and remain epidemiologically realistic, aetiological agent and transmission mode subgroups were not constrained to be mutually exclusive (e.g., a disease can be both vector-borne and zoonotic, such as zoonotic malaria).

We also provide the full dataset for readers to ensure transparency and reproducibility.

Randomization

As this was a systematic review of empirical observational studies, no randomization could be conducted. However, we aimed to generate as homogenous of a dataset that could be conducted given the paucity of published data. This was done through our strict inclusion and exclusion criteria. In addition, we also aimed to deal with the issue of heterogeneity, confounding and effect modification through our subgroup analysis which was conducted using a priori subgroups and using the E Test.

Blinding

Blinding of participants was not possible as all included studies in the review were either case control or cross sectional. Blinding of interviewers or survey conductors was not conducted or reported for any of the studies.

Did the study involve field work? ☐ Yes ☒ No

# Reporting for specific materials, systems and methods

We require information from authors about some types of materials, experimental systems and methods used in many studies. Here, indicate whether each material, system or method listed is relevant to your study. If you are not sure if a list item applies to your research, read the appropriate section before selecting a response.

## Materials & experimental systems

| n/a | Involved in the study |
|---|---|
| ☒ | ☐ Antibodies |
| ☒ | ☐ Eukaryotic cell lines |
| ☒ | ☐ Palaeontology |
| ☒ | ☐ Animals and other organisms |
| ☒ | ☐ Human research participants |
| ☒ | ☐ Clinical data |

## Methods

| n/a | Involved in the study |
|---|---|
| ☒ | ☐ ChIP-seq |
| ☒ | ☐ Flow cytometry |
| ☒ | ☐ MRI-based neuroimaging |
