# Supplementary material for: Agricultural land-uses consistently exacerbate infectious disease risks in Southeast Asia
Source: Nat Commun. 2019 Sep 20;10:4299. doi: 10.1038/s41467-019-12333-z (PMC6754503; doi:10.1038/s41467-019-12333-z)
Supplement: Supplementary file 4 — Description of Additional Supplementary Files [file 41467_2019_12333_MOESM4_ESM.pdf]

Description of Additional Supplementary Files

File Name Supplementary Information Dataset 1

Description: Supplementary Data 1 gives the raw data used in all analyses. Below is a table which is a data key that describes the type of data in each column.

Column Name	Variable Description
Author	The name of the first author of the study.
Year	The year the study was published.
Rural/Urban	Whether the study location was rural or urban. Responses can be Rural, Urban or National Level Data.
Children in population	Whether children were included in the total population. Binary response of Yes or No.
Gender breakdown	What was the gender breakdown of the population? Majority Male = more than 60% of the population was male. Majority Female = more than 60% of the population was female. Equal = 50% of the population was male and 50% was female.
Country	The country the study was conducted in.
Sampling type	The type of sampling conducted. Random denotes random sampling. Purposive denotes purposive sampling.
Outcome measurement	How was prevalence, seroprevalence or incidence measured. This is a binary variable which could be laboratory or clinical. Laboratory = outcome is measured using a laboratory test. Clinical = the outcome is measured using a clinical diagnosis.
Study type	The type of epidemiological study conducted.
Study Quality	This is based on the National Heart, Lung, and Blood Institute (NHLBI) study quality tool for case control and cross-sectional studies. Studies are evaluated as Good, Fair or Poor.
Exposure	This is the type of agricultural exposure.
Comparator	The type of comparator
Disease	The type of disease
caseexpyes	The total number of people who are exposed and are infected by a pathogen.
caseexpno	The total number of people who are exposed and are not infected by a pathogen. (Cell C in the OR framework below)
controlexpyes	The total number of people who are not exposed and are infected by a pathogen. (Cell B in the OR framework below)
controlexpno	The total number of people who are not exposed and are not infected by a pathogen. (Cell B in the OR framework below)

Log odds ratio	The log of the crude odds ratio
Sampling variance	Spread or variability of the sample estimate
Bacterial	Binary response on whether the disease can be classed as a Bacterial Agent
Parasite	Binary response on whether the disease can be classed as a Parasitic Agent
Vector-Borne	Binary response on whether the disease can be classed as a Vector-Borne Agent
Zoonotic	Binary response on whether the disease can be classed as a Zoonotic Agent
Virus	Binary response on whether the disease can be classed as a Viral Agent
Regional meta-analysis	Binary response which indicates which studies were included within the regional meta-analysis
Study Characteristics Subgroups	Binary response which indicates which studies were included within the study characteristics subgroup analysis
Exposure Based Subgroups	Binary response which indicates which studies were included within the exposure-based subgroup analysis
Disease Based Subgroups	Binary response which indicates which studies were included within the disease-based subgroup analysis

<u>OR FRAMEWORK</u>

<table>
<tr><td colspan="2" rowspan="2">OR Framework</td><td colspan="2">Diseased/Infected</td></tr>
<tr><td>Yes</td><td>No</td></tr>
<tr><td rowspan="2">Exposed</td><td>Yes</td><td>caseexpyes</td><td>caseexpno</td></tr>
<tr><td>No</td><td>controlexpyes</td><td>controlexpno</td></tr>
</table>

Here is an example of the framework used to calculate crude odds ratios.
